# Shifting care from hospitals to general practice from the health insurers’ perspective: an interview study

**DOI:** 10.1186/s12913-025-13650-4

**Published:** 2025-11-20

**Authors:** Jesper T. Dros, Isabelle Bos, Christel E. van Dijk, Bert R. Meijboom, E. J. E. van der Hijden, L. Timmers, Robert A. Verheij

**Affiliations:** 1 Netherlands Institute for Health Services Research (Nivel), Utrecht, The Netherlands; 2 National Health Care Institute, Diemen, The Netherlands; 3 Tilburg School of Social and Behavioral Sciences, Tilburg University, Tilburg, The Netherlands; 4 Talma Institute, Vrije Universiteit Amsterdam, Amsterdam, The Netherlands; 5 Zilveren Kruis Health Insurance, Zeist, The Netherlands; 6 Department of Health Economics, Erasmus School of Health Policy & Management, Rotterdam, The Netherlands

**Keywords:** Health insurers, Substitution of care, General practice, Hospital care, Reimbursement of care

## Abstract

**Background:**

Policymakers have embraced substitution of hospital care to more affordable primary care as a means to contain rising healthcare costs and provide care closer to home. Health insurers play an important role in the extent to which substitution of care takes place. This study explores the perspective of Dutch health insurers on barriers and facilitators to facilitate a shift from hospitals to general practice in the current healthcare system.

**Methods:**

Semi-structured group interviews were conducted with healthcare purchasers from various health insurers, involving fifteen participants from seven insurers representing 76.5% of the market. Thematic analysis was used to identify perceived facilitators and barriers for effective substitution of care.

**Results:**

Long-term contracts that enable strategic planning and collaboration between general practices and hospitals, as well as strong organizational structures in general practice and long waiting times in hospitals are reported to facilitate substitution. Uncertainties around collaboration under the Competition Act, inadequate compensation through the risk equalization model, complex billing codes for innovative initiatives, a rigid national budgetary framework and strong bargaining power of hospitals as opposed to insurers and general practices are stated to hinder the shift towards general practice.

**Conclusions:**

Key areas for improvement to facilitate substitution, as reported by healthcare purchasers, include clear guidelines on insurer collaboration, adjustments to the risk equalization model, strengthening the bargaining power of general practices, and promoting long-term contracts. This study provides insights into the perceived barriers and facilitators for care substitution from the payer’s perspective. Addressing these barriers is essential for facilitating the shift from hospital to general practice care. Also, potential discrepancies between perceptions and current regulations highlight areas where enhanced dialogue and collaboration between policy makers and health insurers could improve mutual understanding and regulatory compliance.

**Supplementary Information:**

The online version contains supplementary material available at 10.1186/s12913-025-13650-4.

## Introduction

Health policy measures in western countries are increasingly focusing on substitution of care; shifting care from expensive hospitals to more affordable primary care [1–11]. The policy theory is that primary care could potentially deliver care at lower costs and closer to home, while maintaining the same quality and accessibility [12]. While substitution of care has long been promoted in policy, its implementation has proven highly complex [13–16]. Structural factors such as misaligned financial incentives, behavioral resistance among providers and patients and organization of care around traditional pathways have impeded progress [17–22]. Given that health insurers in the Dutch healthcare system are mandated to act as prudent buyers of care, their perceptions and strategies are crucial to understanding the practical feasibility of substitution. This study therefore addresses a critical gap by analyzing how healthcare purchasers from Dutch insurers perceive the barriers and facilitators to shifting care from hospitals to primary care, thereby providing insights into the conditions under which substitution policies can realistically be advanced.

### Background

The Dutch healthcare system consists of a multi-payer system with many private providers. Healthcare providers are contracted by health insurers and are expected to compete for providing effective and efficient care of high quality [23]. While contracts for general practitioner (GP) care are established by the region’s largest insurer and typically adopted by other insurers, hospital care contracts are negotiated individually between each insurer and hospital. The general practitioner (GP) is paid through a mix of capitation and fee-for-service and acts as gate keeper for more specialized healthcare providers. The GP is the central point of contact for health care. Patients are registered at a specific GP practice and need a referral from their GP before visiting medical specialist care. Specialized medical care is financed by Diagnosis Treatment Combinations (DTCs), which has similarities with the DRG system in the United States [24]. Even though the DTC classification is carried out on a national level, most DTC prices are freely negotiable between health insurers and providers [24]. Other providers are often paid through a fee-for-service scheme.

Dutch citizens are policy holders and are able to make active decisions regarding their healthcare providers, insurers and insurance plans, since they have ample choice among providers [25]. Health insurers bear financial risk for the cost of healthcare services provided to its policyholder. Thereby, they are encouraged to reimburse effective and efficient care, which is enforced by competing for policy holders. Dutch health insurers are obliged to enroll all citizens that apply for health insurance, and in return they receive a financial compensation for the predictable healthcare-related costs of their insured population through risk equalization. Without accurate risk equalization insurers are confronted with incentives for risk selection. The financial compensation deriving from the risk equalization model is in addition to a community-rated premium which they can determine themselves. Risk selection can be attractive because premiums are community-rated. The role of health insurers ensures access to care and sustainability of the healthcare system [23]. A more detailed description of the roles, requirements and regulations of Dutch citizens, healthcare providers and health insurers under the Health Insurance Act (HIA) – under which curative care is regulated – can be found in Appendix I. An overview of the HIA relative to the Dutch healthcare system as a whole can be found in Appendix II.

In the Netherlands, steering substitution through supply and demand of healthcare is facilitated by the government by giving patients, healthcare providers and health insurers the right incentives [25]. The government sets boundaries for collectively funded healthcare and preconditions under which these actors can function [12]. Effective steering of these entities is essential to promote the substitution of care from hospitals to general practices, which is seen as a key strategy for improving access, efficiency, and cost-effectiveness within the healthcare system. Several regulatory bodies are instrumental in facilitating this shift. The National Health Care Institute has two instruments through which it can influence substitution of care: (1) advising the government regarding the content of the health insurance package, (2) executing the risk equalization in the care sector. The Dutch Healthcare Authority can enable or restrict substitution by the introduction of billing codes under which healthcare providers are paid by insurers. Also, it can ensure that contractual innovation (e.g. for integrated care or shared savings) complies with existing laws. The Dutch Healthcare Authority also plays a role in supporting transparency and monitoring, and counteracting significant market power, which are important to enable substitution. Additionally, the Authority for Consumers and Markets (ACM) plays a crucial role in ensuring that competition within the healthcare sector remains fair and effective. The ACM clarifies what types of collaboration are allowed, helping general practitioners, specialists, and other actors to work together legally in support of substitution. Also, it prevents market concentration and anti-competitive agreements between hospitals and insurers that could block emerging primary care providers from taking on more services.

### Knowledge gap

Previous research also shows that financial incentives influence both patients’ and healthcare providers’ behavior regarding care utilization. Patients can be incentivized to receive certain types of care, for instance through alterations in care included in the basic health insurance or through exemption from copayments like the otherwise mandatory deductible [21, 26–31]. Healthcare providers can also be incentivized to (not) provide certain types of care. De Vries et al. (2019) found that misaligned incentives in the hospital setting can be a barrier for substitution [32]. Their study reports that volume-based reimbursements installed in hospitals do not stimulate to reduce production in order to substitute care towards the primary care setting, because medical specialists are incentivized to provide more services by the Fee-For-Service (FFS) structure. A way to reduce volume of care is by reimbursing care through salary or capitation instead of on a FFS basis [25, 33, 34]. A way to increase volume elsewhere is by increasing workforces through reimbursement in places where substitution is desired, for example the reimbursement of more fulltime-equivalent mental health nurses in general practice [35].

Health insurers in a multi-payer system could be incentivized to reimburse certain types of care. However, only few studies on the incentives for health insurers have been conducted. One known factor that could incentivize insurers to reimburse certain types of care is risk equalization [16–37]. The risk equalization model could form a barrier for health insurers to facilitate this substitution. Health insurers are compensated for the risk of their ensurees through risk equalization. The risk equalization model consists of age and gender groups, source of income, socio-economic status, persons per address, region and chronic diseases. These chronic diseases are based on provided healthcare services through pharmaceutical cost groups, diagnostic cost groups, medical devices groups, multiple-year high cost groups, physiotherapy diagnoses groups and home nursing groups [38]. Initiatives aimed at shifting care from hospitals to primary care settings reduce the volume of hospital-based healthcare services. Important initiatives are for example integrated care for chronic conditions with high prevalence like COPD, cardiovascular diseases and diabetes type 2, in which care is shifted from hospitals towards primary care for large populations of patients [22, 39]. Since most primary care services do not directly affect the compensation for chronic diseases, this leads to less patients for which chronic diseases are compensated. Therefore, substitution could lead to a lower average compensation for health insurers, which in turn may lead to a lower net financial result. In case of lumpsum contracts for a hospital, the business case for an insurer may decrease further in the short term [40–44].

### Aim

Substitution of care has been extensively studied from the perspective of providers [1, 4, 18, 19]. To our knowledge, comprehensive studies regarding the factors (e.g. organizational, financial, regulatory. relational) considered by health insurers in facilitating substitution have not yet been conducted. Therefore, we aim to gain more insight into these factors by examining the perceived barriers and facilitators for substitution between general practitioners and medical specialists by health insurers. It is important to note that despite its theoretical appeal, substitution has seen limited implementation across Western healthcare systems. This indicates the presence of both system-specific and more universal barriers and facilitators, highlighting the relevance of this topic beyond individual healthcare systems.

## Method

### Design and sampling

Given the complexity and context-specific nature of healthcare purchasing decisions, we chose a qualitative method to capture nuanced insights, attitudes, and the reasoning behind purchasing behaviors that quantitative methods may overlook. Semi-structured group interviews were conducted with healthcare purchasers of both general practice care and hospital care, working at various Dutch health insurers. To ensure a broad understanding of healthcare purchasers’ experiences, we invited all Dutch health insurers through email, in which we explained the rationale for the study. In 2023, ten health insurers were responsible for healthcare reimbursement in the Netherlands (four large insurers and six small insurers). Many of these health insurers have several subsidiaries operating under different brand names (20 subsidiaries in total) [45, 46]. The four largest health insurers have a combined market share of 85.1% [47].

### Data collection and setting

In seven group interviews (one per insurer), we interviewed fifteen employees working at seven health insurers in the Netherlands (three large and four small insurers). We interviewed at least one purchaser of GP care and one purchaser of hospital care per insurer. Two health insurance companies declined our request for participation, citing time constraints, while one health insurer did not provide a response. Interviews were conducted by the primary researcher (JD, MSc, PhD student), either alone or together with IB (senior researcher, PhD), RV (program leader, Prof. dr.) or CD (coordinating advisor, PhD). Interviews were held either online or in person, depending on preference of the insurer. In order to gain insight into incentives experienced by health insurers, healthcare purchasers were deemed as most appropriate employees to interview since this is their daily task. After five interviews with three large and two smaller health insurers, data saturation was reached. However, two additional interviews with smaller health insurers were held to ensure actual saturation of the data. All respondents were actively engaged in healthcare purchasing, ranging from operational to strategic level. The experience of the respondents within the health insurance sector ranged from less than 1 year to more than 25 years of experience. The health insurers included in our study had a combined market share of 76.5%, based on the publicly available market share per health insurer [48].

A topic list was constructed based on previous studies regarding the role and incentives of health insurers in the Netherlands [32, 36, 37, 42–44], informal interviews among employees of the Dutch Ministry of Health, Welfare and Sports, the National Health Care Institute, several (former) employees of health insurers and discussions within the research group (appendix III). Also, responses from interviews were used to update the topic. First, we explained the background and aim of our study. Field notes were made during interviews and discussed afterwards between researchers. Interviews were held between February and April 2023 and took about 60–90 min each. All interviews were audio recorded and transcribed verbatim. De-identified transcripts were made by an external agency and were then sent to the respondents afterwards for review and approval. All respondents provided their consent.

### Data analysis

Transcripts were analyzed by two researchers (JD & IB), following the steps of thematic analysis [49]. For the initial transcript, we familiarized ourselves with the data, generating initial codes. Then, we compared our initial codes and searched for emerging themes. We applied thematic analysis with a primarily inductive approach to allow themes to emerge naturally from the participants’ accounts, rather than imposing preconceived categories. This approach was selected because the perspectives of Dutch healthcare purchasers on substitution of care have not been extensively studied before, making it important to remain open to unexpected insights. Discrepancies were discussed until consensus was reached. Following, we named and defined themes. The consecutive transcripts were analyzed by JD, of which IB checked transcript 2 and 6. Discrepancies were discussed again until consensus was reached, and other transcripts were adapted accordingly. Thematic analysis was used because this is a flexible and systematic method for identifying themes within qualitative data [49]. Themes were partly identified in advance through existing literature, and complemented with emerging themes from the data. The coding tree can be found in Appendix IV. Transcripts of each interview as well as an overview of our findings were shared with participants to enable them to give their feedback. We used MAXQDA version 24 in the coding process.

### Research team and reflexivity

Both JD and RV had previous experience with qualitative research. JD and IB followed a qualitative research course in preparation for this study provided by H. Boeije, who lectured and wrote several books on qualitative research [50]. The interviewers have no prior assumptions to report. The Consolidated Criteria for Reporting Qualitative research (COREQ) checklist can be found as a separate file in the supplementary material [51].

### Ethical considerations

The study adhered to the principles outlined in the Dutch Personal Data Protection Act. Participants’ anonymity was rigorously maintained throughout the study. Additional ethical approval was not required under Dutch law, as all participants were competent adults, were not subjected to any procedures, and were not required to follow specific behavioral guidelines (for details on Dutch legislation, see: http://www.ccmo.nl/en/). We did not provide participant numbers, because this would lead to traceability and therefore privacy issues due to the small number of existing Dutch insurers.

## Results

Health insurers are familiar with substitution of care. Many of them participate in so-called regional data coalitions, in which they monitor substitution and evaluate its progress. With increasing digital options like online joint consultations between GPs and medical specialist for instance, the possibilities to facilitate substitution have increased, which could serve as a stepping stone to facilitate substitution. However, several barriers and facilitators are reported by healthcare purchasers, which are presented by theme below in Table 1 and will be elaborated consequently.

**Table 1:** Factors influencing substitution as reported by healthcare purchasers

Theme	Subtheme	Description
(Mis)alignment of stakeholders on substitution		Mutual agreement between general practices and hospitals of when substitution is appropriate, is reported as an important facilitator by healthcare purchasers.
Financial incentives	Long term contracts	Long term contracts are reported to be a facilitator as they ensure structural income. Not all purchasers are welcoming structural reimbursement (S1*) in general practice though, as they give way control.
	Risk equalization	Some healthcare purchasers report that risk equalization can disincentivize shifting patients from hospitals to general practice, prompting concerns. Other purchasers see no barriers of risk equalization.
Organization of care	Organization of hospitals	Hospitals with long waiting times have to be selective in which patients they treat, and which patients could be treated by the general practitioner, which facilitates substitution. In contrast, other hospitals might want to produce a certain volume of care to remain financially viable. Other reported barriers are layering of management within hospitals, and bargaining power of hospitals.
	Organization of general practices	Organizational power of GPs varies regionally. Less bargaining power as opposed to hospitals and a lack of urgency of substitution are reported as barriers for substitution.
National policy	National budgetary framework	Many healthcare purchasers are bound by the national budgetary framework and find that it doesn’t incentivize substitution, because it does not shift along with substitution. Some small insurers’ purchasers report they are not bound by the national budgetary framework.
	Billing codes for reimbursement	Filing for new billing codes sometimes become so complex that it is difficult to work with them, which hinders agreements on substitution initiatives.
Collaboration versus competition		Healthcare purchasers report it to be unclear in what extent they can collaborate in order to facilitate substitution under the Competition Act.

*= structural reimbursement of general practice care is carried out from segment 1 (S1), which is explained in detail in the results section of this paper

## (Mis)alignment of stakeholders on substitution

According to healthcare purchasers, medical specialists and general practitioners do not always agree on when substitution is appropriate. This is reported by purchasers as more problematic than the actual reimbursement: *“They [medical specialists and general practitioners] first need to agree on whether this should be done in primary care or secondary care […] Once they agree on that*,* nine times out of ten*,* we can then figure out together how to finance it.”*

Mutual understanding of when substitution is appropriate, is reported as an important factor by healthcare purchasers. Strong regional collaboration is also regarded as an important factor between all parties involved (general practitioner, medical specialist and health insurer): *“This may sound cliché*,* but you need trust and a sort of mutual goodwill towards each other. If you don’t know each other*,* it’s much harder to achieve that.”*

According to this healthcare purchaser, parties should set aside their own ego in order to work together for the best result: *“We need to find a way for organizations to look beyond their own egos and ask: Can we ensure that we*,* as a region*,* can handle this together*,* rather than whether I achieve my budget and results? Instead*,* we should focus on delivering healthcare as a region.”*

## Financial incentives

Financial incentives are reported by healthcare purchasers to play a large role in the extent to which they can facilitate substitution of care.

### Incentives for providers

Hospitals need to produce a certain volume of care to remain financially viable. When hospitals are financially unhealthy, healthcare purchasers actually have to reverse or prevent substitution in order for the hospital to remain viable: *“If you talk about shifting from secondary care to primary care*,* for example*,* you actually don’t want that [for unhealthy hospitals] […] because you want to maintain the infrastructure of the hospital. So*,* I want to bring care inward. If you then also start moving care to primary care*,* you’re actually emptying them out again. So*,* from a reimbursement perspective in our role… That’s very strange there. You’re making a contradictory move from what you should or would like to do.”*

One of the ways to ensure viability of hospitals, is through long term contracts. Contracts between insurers and providers are often annually revisited. Longer term contracts are reported to facilitate substitution, as they ensure providers of structural income, even when care is shifted away from them. Hence, this is a positive financial incentive to support substitution. Most healthcare purchasers report to make multi-year agreements with hospitals, in order to facilitate them in the transition needed for substitution. An example: *“[…] So*,* we made agreements with the hospital*,* a multi-year agreement – of ten years – which includes a lump sum. We also made agreements with the general practitioners in which we say: we agree on referring less [to the medical specialist]. And what’s important in that*,* in shifting care*,* is that you also include the financial consequences in that story. So if we could demonstrate: those general practitioners referred*,* let’s say*,* one million euros less*,* then we want to see that reflected in the lump sum*,* in how that lump sum is calculated*.”. In this example, the lump sum incentivizes hospitals to provide care efficiently. At the same time, this lump sum agreement can be altered when referrals towards the hospital are reduced.

In general practice care, the nature of contracting is also reported to play a role, which can be a barrier for substitution. General practice care reimbursement is divided into three segments. Segment 1 (S1) consists of basic provision of general practitioner care, with a combined system of fee-for-service and capitation. This includes, among others, consultations with the general practitioner, mental health practice nurse (POH-GGZ), and consultations for transient patients. Segment 2 (S2) consist of programmatic multidisciplinary care, which involves additional disciplines to general practitioner care. There must be an agreement with the relevant health insurer to be able to bill for services from this segment. These agreements can be practice-specific. Segment 3 (S3) consists of performance-based rewards and care innovation. In this segment, there is room for health insurers and general practices to make agreements about the outcomes of general practitioner care and multidisciplinary care. This segment also allows for practice-specific agreements regarding general practitioner care or multidisciplinary care. The underlying idea is that, when these experiments prove successful—through research, monitoring, or practical experience—they can be transferred to S1 or possibly S2, which represent the more structural funding arrangements within general practice. Key focus areas include substitution, e-health, and joint consultations. A contract is also required for the third segment.

Insurers report that innovative initiatives that facilitate substitution between hospitals and general practice mostly start locally and are reimbursed in S3, were tailor made agreements can be made. When initiatives are successful, the upscaling process from S3 towards structural reimbursement in S1 is reported to be challenging. The Dutch Healthcare Authority has to authorize the initiative and its adjacent reimbursement code, which then means that all health insurers are obliged to reimburse these initiatives for all contracted providers under predefined conditions. This ensures structural income for providers, but is not always preferred by healthcare purchasers: *“I believe that as an insurer*,* you naturally have many more tools and leverage when you have the flexibility to make tailored agreements. So*,* our standpoint as insurers is: don’t put everything into S1*,* because you will lose the tools to make effective agreements in your region. Precisely when you can offer the comfort within those tailored agreements of not doing it for just one year*,* but for a longer period*,* then you can reach an agreement together. […] as long as you*,* as an insurer*,* are a reliable partner and don’t undermine every agreement after a year or two.”*

Another healthcare purchaser is concerned that including these initiatives in S1 might give an incentive to provide more care than necessary: *“We also see the downside. If you set a fixed reimbursement code […] then you also can’t manage it anymore. It can also have a huge volume incentive effect. […] any GP can use it for any specialty. Whereas now […] you make agreements with each other about it. For which disciplines yes or no? Is some specific training required before you do it? Do you have to align with regional collaboration agreements?”*

### Incentives for insurers

Experiences from healthcare purchasers regarding the risk equalization are mixed. While some respondents knew little about it or did not report any barriers from risk equalization towards substitution, other insurers did report incentives from the risk equalization: *“You sometimes see that as long as a patient is in secondary care*,* they generate more revenue for the insurer than they would cost if they moved to primary care […]. We’ve also encountered this issue with some business cases. When we looked at the costs of patients in secondary care and the revenue from risk equalization*,* moving them to primary care even resulted in a negative outcome. So*,* should we move those patients? This wasn’t an incentive for us to avoid doing it*,* but it was a reason for us to raise the issue at a higher level and say: listen*,* something is not right here”*.

Another healthcare purchaser reported the same barrier, in a specific case: *“A lot is at play here. With the shift towards general practitioners… We conducted a pilot with specialists. For example*,* a Diabetes Expertise Center or a Cardiovascular Expertise Center*,* where the hospital primarily assessed cases. That center had two sides: general practitioners could consult specialists for advice on whether they could continue treating a patient themselves*,* and at the hospital*,* they also evaluated which patients were unnecessarily in secondary care and could be referred back to primary care. These specialists were also part of this process—it was simply part of the operation*,* with shared objectives. This approach worked positively*,* helping to reduce unnecessary referrals. However*,* sometimes we face challenges related to the risk equalization system in the background. In some cases*,* as long as a patient remains in secondary care*,* they generate more revenue for the insurer than they would cost if transferred to primary care. That’s an issue*,* but efforts are being made to address the fact that the system is not always perfectly balanced.”*

## Organization of care

### Organization of hospitals

According to insurers’ healthcare purchasers, the (lack of) workforce capacity in hospitals, gives hospitals an incentive to keep or hold back certain care. Due to long waiting times, hospitals have to be selective in which patients they treat, and which patients could be treated within general practice. This is also what one healthcare purchaser reports: *“Meanwhile*,* the tide is turning*,* as hospitals are facing staffing shortages. So*,* they have to start selecting at the gate who they really need to help and who can be assisted by the general practitioner. And then you see that the selection mechanism also works better for themselves”.*

Healthcare purchasers stated that they experience difficulty to shift care provision away from hospitals in particular due to layering in the managements of hospitals and to make the shift from agreements on paper to plans in practice. “*You don’t always speak with the [doctors in the] hospital directly*,* so you’re dealing with someone from the hospital*,* I don’t know*,* a director or someone in the staff who says*,* ‘That’s a great idea*,* we’ll do that.’ But that doesn’t mean that you always have the entire department*,* the specialists*,* on board. So that makes it difficult again in such a hospital dynamic.”*

Bargaining power of hospitals relative to general practices is also reported to withhold the shift towards general practices. While hospitals can be fragmented within their organization – as illustrated in the previous quote – they can have more bargaining power once consensus is reached, as compared to relatively small general practices. “*Hospitals generally have more leverage and are often larger*,* so they tend to be more proactive and eager to get things moving quickly. We want to avoid a situation where insurers can make an agreement with the hospital separately from the agreements made with the GPs. We’ve stressed the importance of joint efforts and ensuring that there’s impact on both sides*,* to prevent potential disagreements.”*

### Organization of general practices

General practices are smaller than hospitals and are therefore often organized in healthcare groups for regional collaboration. However, these healthcare groups are often more fragmented than hospitals. Therefore, they might have less bargaining power and it takes more effort to align and reach consensus. Since substitution is a joined effort, this could form a barrier: *“In [region]*,* everything the healthcare group comes up with must first be presented to its members [individual general practices]. There’s a lengthy back-and-forth discussion about it*,* and then they may or may not get approval. So progress is very slow*,* moving forward in tiny increment”.* And while purchasers do report to see enough budgetary room for substitution, they see less organizational power at general practices.

Fragmentation of general practice care is not problematic in all regions. One insurer reimburses care in a region where general practices are really well organized, which directly translates in better bargaining power towards insurers: “*If you look at [healthcare group]*,* they have developed a concept where they negotiate with [insurer] on behalf of all general practitioners regarding a portion of the funding. […] So*,* in those practices that are affiliated with them*,* there is a significant mandate and broad support.”*

## National policy

### National budgetary framework

The national budgetary framework dictates the annual healthcare budgets per sector [52]. Healthcare purchasers report that general practitioners often ask them why the budgetary framework does not shift from specialized medical care towards general practice care, along with the shift in care that is desired. While some purchasers from small insurers report that they are not bound by the national budgetary framework since they reimburse only a fraction of the nationwide curative healthcare costs, most healthcare purchasers report that they are bound by this framework and that it does not incentivize them now to facilitate substitution: *“We can’t just say*,* ‘We have a total of 100 million*,* and let’s allocate 30/70 for GPs/hospitals in 2023*,* and then shift to 40/60 in 2024*,* because we’re facilitating that movement [substitution]. We have earmarked funds specifically for GP care*,* and we need to consciously allocate them accordingly. But it’s not very encouraging because we still have those rigid barriers in place. It’s not like we’re breaking them down and reallocating a significant portion elsewhere*,* which would make more sense.”*

In reality, general practice care has had an underconsumption of their available budget for many consecutive years [53]. Therefore, it would seem that budget is not the problem. However, regional variation is important, as is reported by one health care purchaser: *“At the national level*,* you can indeed say that [there is underconsumption of the available budget]*,* but when you cascade it down to the insurers*,* then to a regional organization*,* and below that to a general practitioner*,* it can vary by region. So*,* in one region*,* you might say: oh yes*,* we might still have some financial room left. But there are also regions where we’re already putting in much more financial resources*,* so that’s not the case there”.*

Additionally, many health care purchasers report that the growing shortage in general practice care also makes it challenging to allocate the entire budget. Funds cannot be spent on providers who are not available.

### Billing codes for reimbursement

Besides the national budgetary framework, there is also an experienced gap between national healthcare policy and practice. For instance, in the design of reimbursement methods that stimulate specific consultations to be introduced in general practice to facilitate substitution: *“I think it’s again that gap between—not to speak ill*,* that’s not the intention—but the gap between policymakers*,* let’s call it somewhat disrespectfully*,* and the floor*,* so what’s really happening. I think that gap is so large that from good intentions the idea comes: let’s create a reimbursement code for it. But in reality*,* it doesn’t match at all with the needs.”*

As a result, filing for new billing codes sometimes become so complex that it is difficult to work with them, which hinders agreements on substitution initiatives: “*We’ve told the Dutch Healthcare Authority that the whole process [of filing for a new billing code to be able to finance care that hasn’t yet been established in legislation and regulations] is quite a monster. So that really creates a barrier*,* also for us as insurers to finance innovation through that route. […] You just have to provide a lot*,* fill in monitoring agreements via formats. That’s just a complicated process. And maybe that’s again because we’re small. So that really burdens your workload.”*

## Collaboration versus competition

Healthcare purchasers report it to be unclear to what extent they can collaborate, and when they are not allowed to collaborate under the Competition Act. Therefore, one healthcare purchaser proposed a collaboration framework, in order to determine when collaboration is allowed under the Competition Act: *“There needs to be a solid framework for determining when collaboration is appropriate and when it is not. Essentially*,* I believe the guideline should be that all insurers are competitive*,* unless there’s an exception. […] From that exception*,* you can then specify which areas are suitable for collaboration and which are not.”*

When this uncertainty leads to a lack of collaboration, healthcare purchasers stated that the risk of double funding arises, as they are unaware of certain substitution agreements made between hospitals and other health insurers. This occurs because healthcare purchasers may not be fully informed about all existing agreements made by other insurers, leading to overlapping or duplicate funding for the certain care services: *“A major risk that arises […]*,* is the potential for double funding. If in another region*,* specific agreements are made with general practitioners for hospital-shifted care*,* and we are not fully aware of [the adjacent agreements in the hospital]*,* we will not adjust our agreement with the hospital accordingly because we don’t know the details. So*,* it could be that market leaders have made certain shifts that other insurers haven’t. This is always a danger and a challenge that we all*,* because everyone has this*,* need to be vigilant about.”*

## Discussion

Our aim was to gain insight into barriers and facilitators to facilitate a shift from hospitals to general practice in the current healthcare system, from the perspective of Dutch health insurers. We found that, as perceived by healthcare purchasers in the Netherlands, a range of factors influence substitution of care, including both barriers and facilitators. While a broad range of barriers and facilitators emerged, we will focus here on those themes that appear most influential in shaping the implementation of care substitution by health insurers. Facilitators included long-term contracts between insurers and healthcare providers, which enable better planning and upscaling of care. Strong collaboration between general practices and hospitals, supported by digitalization, joint e-consultations and data coalitions also stimulate the shift of care to general practices. Long waiting times in hospitals that necessitate selective patient treatment also enforce substitution towards general practices. Barriers included the ambiguity of the Competition Act regarding insurer collaboration, hospitals’ bargaining power, and the current risk equalization system.

Sometimes there seemed to be a discrepancy between perceived barriers and facilitators and the current practical implementation of laws and regulations. However, these perceptions are crucial for the extent to which substitution actually occurs. They often arise due to the complexity of the healthcare system, making it essential to actively identify and address these perceptions to remove perceived barriers to substitution. Discrepancies in perception and the current practical implementation of laws and regulations perhaps make experiences from healthcare purchasers even more relevant. When healthcare purchasers perceive laws and regulations as a barrier, this might as well be a self-fulfilling prophecy, even though policymakers are convinced that substitution is not disincentivized on paper. Perceptions of reality, influence this reality. Or in the words of sociologists William and Dorothy Thomas: “If men define situations as real, they are real in their consequences” [54]. An example of discrepancies in perception and the current practical implementation of laws and regulations is a commonly reported barrier regarding the tension between collaboration and competition. Under the Competition Act [55], health insurers are only allowed to collaborate to a limited extent. Healthcare purchasers stated that these limitations to collaborate hinder substitution. The Competition Act prohibits collaboration if they have the intention to or will result in hindrance, impediment or distortion of competition on the Dutch healthcare market. However, according to the Authority for Consumers & Market (ACM), certain forms of collaboration are acceptable in a system of managed competition if the benefits of collaboration outweigh the necessary restrictions on competition [56]. While guidelines from the ACM for collaboration between insurers exist [57–59], insurers still perceive the Competition Act to be unclear as to what extent they can really collaborate. This has been reported in the literature before, in the context of collaboration between insurers to ensure quality of care [56]. To facilitate substitution, it is essential to bridge the gap between healthcare purchasers’ perceptions of appropriate collaboration and what is permitted under the Competition Act.

Besides the ACM, the National Health Care Insititute (NHCI), the Dutch Healthcare Authority and the Ministry of Health, Welfare and Sports could play an important role in addressing barriers for effective implementation of substitution, as reported by healthcare purchasers. A significant challenge lies in the alignment of perspectives from general practitioners and medical specialists on when substitution is appropriate. Alignment of these perspectives is reported to be challenging due to multiple layers of management and decision-making within hospitals. The challenges highlight the importance of clarifying when substitution is appropriate. The NHCI could play a role here by fostering consensus between general practitioners and medical specialists and providing clear guidance for when substitution is appropriate, while keeping the regional context in mind. Another challenge is the procedural complexity of filing for billing codes is reported to hinder substitution initiatives. The Dutch Healthcare Authority could play an essential role in simplifying the procedures for the application of these codes, streamlining the way for providers to engage in substitution. Lastly, the Ministry of Health, Welfare and Sports could communicate and clarify the influence of substitution on risk equalization for insurers, since some healthcare purchasers report the risk equalization to be a barrier for substitution.

Perceived incentives deriving from the current risk equalization model, as found in previous studies [16–37], are confirmed by healthcare purchasers in our study. Potential solutions to mitigate this problem as proposed in earlier studies, are:

1. By not directly linking equalization features to healthcare utilization in specific sectors like hospitals or general practices, but instead categorizing by (chronic) condition, incentives to keep patients in more specialized care are diminished;
2. Adding primary care services to equalization criteria. While this could be beneficial, there are concerns about potential unintended consequences. Since the costs of primary care are relatively low, including it in the equalization model might lead to undesirable financial incentives. For instance, upcoding from an insurers perspective (artificially inflating patient risk levels to gain more compensation) could become more prevalent. This suggests a need for caution when considering such changes to avoid creating new distortions in the system;
3. Stabilizing the equalization contribution by retaining the characteristic for a chronic condition for a longer period, reflecting the chronic nature of such conditions. This would ensure stable contributions even if healthcare usage shifts to primary care;
4. Tailored solutions for specific cases were insurers are financially hindered if substitution takes place.

All of these solutions have advantages and disadvantages, and have many challenges surrounding feasibility, data availability, efficiency and legal constrains [36, 37]. Our study contributes to the ongoing discussion by reaffirming the perceived incentives that derive from the current risk equalization model. Our findings underscore the challenges healthcare purchasers face in navigating these incentives, particularly in promoting substitution from specialized to primary care. At a minimum, clarification of the influence of substitution on risk equalization for insurers is needed. Note that this does bear a risk; if substitution effects are negative and substantial, but solutions are not feasible, then this might still disincentivize substitution.

Another theme that emerged in our study was the usage of long-term contracts, which confirms earlier studies. As long-term financial planning and commitment are necessary for substitution, contracts with a longer duration are a way to facilitate agreements between general practices and hospitals. There is ample economic literature understating the importance of long-term contracts. By reducing the risk for discontinuation of the contract, the supplier (of healthcare) is more likely to make investments [60]. Of particular importance in healthcare services are dedicated assets: general investments by a healthcare provider that would not be made in the absence of the prospect of selling a significant volume of the product to a specific buyer [61]. If the contract agreement is terminated, the healthcare provider is left with spare capacity. Therefore, investments in dedicated assets (e.g. personnel or treatment facilities) might be withheld under short-term contracts. Bargaining power of general practices towards hospitals was also found to be an important theme for healthcare purchasers. Since hospitals and general practices often decide together which care can appropriately be substituted, bargaining power between these actors plays a large role in what agreements are made.

Significant differences in bargaining power between hospitals and general practices, which consequently influence the shift towards general practice care, are reported in our study. Bargaining power has been found in earlier studies to play a crucial role in the healthcare system with regards to negotiations, resource allocation and the implementation of new healthcare initiatives [62–64]. Hospitals generally possess more bargaining power as compared to general practices, primarily due to their larger size. Gaynor and Town (2012), already asserted in previous research that larger healthcare providers can exert significant influence over insurers and other stakeholders due to their substantial market presence and resource capabilities [62]. General practices on the other hand, are typically smaller and more fragmented, often organized into healthcare groups to facilitate regional collaboration. Healthcare purchasers reported that this can slow down progress and innovation, which is supported by earlier research from Scott et al. (2011), who found that smaller healthcare organizations often struggle with internal coordination [63]. However, when internal coordination is well-organized and general practices are unified, they can enhance their bargaining power and thereby facilitate better resource allocation [64].

Within our study, we observed some important differences between small and large insurers. It is important to note that every insurer is organized differently. All insurers have separate divisions for GP and hospital care. Large insurers also have overarching strategic divisions that link these separate divisions. Smaller insurers often do not, since purchasers reach each other more easily with direct lines of communication. Since small insurers often have limited manpower, they focus on core activities rather than the pursuit of broad-scale initiatives. They often operate regionally; their close collaboration with local healthcare providers allows them to leverage deep regional knowledge, ensuring that their offerings are closely aligned with the specific needs of the communities they serve. Therefore, small insurers might sometimes be better equipped to steer towards substitution of care through local knowledge, networks and their position within the community. Large insurers, on the other hand, operate on a broader scale, enabling them to implement and assess initiatives across multiple regions. Thereby, they can compare and adjust strategies based on diverse regional data. Therefore, larger insurers might sometimes be better equipped to initiate and evaluate substitution initiatives. Each type of insurer offers unique benefits: small insurers focus on localized expertise and close partnerships, while large insurers can develop broad operational capabilities through extensive comparative analyses and insights.

While this study explores the perspectives of healthcare insurers regarding the barriers and facilitators of substitution in the Dutch healthcare system, it is important to view this perspective relative to the position of important stakeholders such as general practices and hospitals. As stated in the introduction, Dutch patients require a referral from their GP to visit medical specialist care. This gatekeeping role is already aimed to channel care more appropriately and may limit the scope for further substitution of hospital care with GP care compared to systems where patients can access medical specialist care directly. However, it also offers the potential to actively steer substitution initiatives, given that GPs are already positioned to coordinate care pathways. There appears to be a shared recognition across stakeholders of the complexity involved in shifting care settings. For example, all parties emphasize the importance of well-defined agreements and collaboration protocols to ensure continuity and quality of care. However, substitution is not only a matter of financial or organizational arrangements but involves deeply interwoven professional roles and dependencies. Both GPs and hospitals discuss the appropriateness of transferring certain tasks [4, 19]. GPs also highlight practical limitations such as high workload [4, 18]. Hospitals, meanwhile, may be concerned about losing patient volume and the associated revenue [32].

### Strengths and limitations

Previous studies investigating the experiences of health insurers, focused on topics like acting as a healthcare advisor, engaging citizens in procurement decision making, the role of health insurers as prudent buyer, or regarding collaboration between insurers in order to ensure quality of care [45, 65, 66]. Our study pioneers on gaining insight into the experiences of health insurers with facilitating substitution.

By conducting semi-structured group interviews with healthcare purchasers from both general practice care and medical specialized care at various Dutch health insurers we were able to capture a broad range of experiences and insights. Also, we interviewed healthcare purchasers from both small and large insurers, enabling us to gather experiences from the perspective of different market positions. Notably, the included health insurers had a combined market share of 76.5% – responsible for a large share of all Dutch inhabitants. The data saturation reached in our study contributes to the robustness of our findings. The use of thematic analyses allowed us to uncover both anticipated and emergent themes in order to gain a comprehensive understanding of the facilitators and barriers experienced by healthcare purchasers in facilitating substitution.

A critical consideration when interpreting our findings is the potential influence of self-serving bias in the responses of participants. Self-serving bias, the tendency to attribute positive outcomes to internal factors while attributing negative outcomes to external circumstances, may have shaped the way healthcare purchasers discussed enablers and barriers to care substitution. Moreover, group interviews could have influenced the responses due to socially desirable answers or more vocal participants, potentially skewing or influencing the results. However, we believe the interdisciplinary subject of our research is best studied with purchasers from both disciplines present, enabling them to complement each other. This was evident from the interviews, as respondents frequently referred to one another to provide more comprehensive and accurate answers to the interview questions. Without group interviews, we would therefore likely have missed out on relevant information. While our study included a large share of all Dutch health insurers, not all insurers operating in the Netherlands were included. Two insurers decided not to participate and one insurer did not respond to our request. This could potentially limit the completeness of our results. However, since data saturation was reached well before all interviews were conducted, we expect this to be of little influence. One of the two insurers that declined, also declared that their input would likely be similar to other insurers in a comparable market position, which is also suggested by the aforementioned data saturation.

## Conclusion

This study uniquely contributes to the literature by providing the first in-depth exploration of Dutch health insurers’ perspectives on the barriers and facilitators influencing substitution of care from hospitals to general practice. Understanding this perspective is vital because healthcare purchasers hold key decision-making power over contracting and reimbursement, which directly affects the feasibility of substitution efforts. Addressing barriers reported by healthcare purchasers is essential for advancing substitution of care in the Dutch healthcare system. Based on the perspectives of healthcare purchasers, some general policy recommendations can be made. The National Health Care institute could foster consensus and provide clear guidance on substitution between healthcare providers. The Dutch Healthcare Authority could simplify the procedures for the application of innovative treatment initiatives, streamlining the way for providers to engage in substitution.

Based solely on the perspectives of healthcare purchasers, it is too early to make more systemwide policy recommendations. Future research could include additional interview studies or focus groups with healthcare providers and policymakers, to provide a complete overview of all stakeholders. Additionally, future studies could more explicitly study the organizational design of health insurers’ purchasing departments, formal governance mechanisms, and cross-functional communication channels between GP and hospital care purchasing departments, as this could potentially enable or discourage substitution. Lastly, the role of governmental bodies in the promotion of substitution, its alignment with current responsibilities of these bodies and the tools at their disposal could also be an interesting subject for future research.

## Supplementary Information

Below is the link to the electronic supplementary material.

Supplementary Material 1

## Data Availability

The data that support the conclusions of this study are available, but restrictions apply to the availability of these data, which were used under license for the current study and so are not publicly available.
