# Supplementary material for: Shifting care from hospitals to general practice from the health insurers’ perspective: an interview study
Source: BMC Health Serv Res. 2025 Nov 20;25:1497. doi: 10.1186/s12913-025-13650-4 (PMC12632074; doi:10.1186/s12913-025-13650-4)
Supplement: Supplementary file 1 — Supplementary Material 1 [file 12913_2025_13650_MOESM1_ESM.docx]

**Appendix I: Roles, requirements, regulatory measures and goals under the Health Insurance Act.**

Table A1-1. Roles, requirements, regulatory measures and goals under the Health Insurance Act.

| **Actor** | **Roles** | **Requirements** | **Regulatory measures** | **Goals** |
| --- | --- | --- | --- | --- |
| **Dutch citizen** | Policy holder | All Dutch citizens are required to purchase basic health insurance. | Uninsured citizens are fined. | Risk solidarity,  No ‘free riding’* |
|  |  | Citizens are free to choose their own insurer. | All Dutch citizens can switch from health insurer once per year at the end of the calendar year. | Freedom of choice |
|  |  | All Dutch citizens are required to pay both a nominal premium and an income-dependent contribution for the basic health insurance. | Contribution is determined in such a way that the total of the income-dependent contributions equals 50% of the total income allocated to the Health Insurance Fund or the health insurers, as specified by ministerial regulation. | Affordability of care,  Income solidarity |
| **Healthcare provider** | Provider of care | Healthcare providers should be able to negotiate a price for their provision of care | For some care types, maximum prices are set by the government. | Freedom of contract |
|  |  | Healthcare providers should provide good quality of care. | Demands for quality of care are set under the Healthcare Quality, Complaints and Disputes Act | Quality of care |
| **Health insurer** | Purchaser of care | Obligation to accept all citizens under their policy, irrespective of their health condition. | Insurers are compensated each year for the risk profile of their policy holders trough ex-ante risk equalization, in which health condition, age, socioeconomic status and other relevant factors are included. This should prevent risk selection behavior from health insurers. Ex-post costs compensation is also applied, when ex-ante risk equalization proves inadequate. | Freedom of choice |
|  |  | Premiums for a policy offered should be equal for all policy holders, regardless of their health condition, age or socioeconomic status. |  | Accessibility of care |
|  |  | Duty of care: obligation to guarantee accessibility of care in the basic health insurance for their policy holders. | The contents of the basic health insurance set by law. | Accessibility of care |
|  |  |  | Selective contracting of healthcare providers is allowed. However, healthcare providers without a contract should also be reimbursed, covering at least 75% of the price. | Efficiency of care |
|  |  | Variations in policies offered are allowed. In principal, possibilities for variation are not limited. | Collective discount was initially allowed, but has been discontinued from 2023 onwards, as it was proven ineffective in reducing costs. | Freedom of policies |
|  |  | The price of policy premiums is set by individual insurers. Each insurer is considered an enterprise within the Competition Act. | Compliance monitoring of the Competition Act is conducted by the Dutch Healthcare Authority and the Authority Consumer and Market | Affordability of care |

Adapted and adjusted from van de Ven et al. (2009) and de Jong et al. (2015). *free riding under citizens is when uninsured ride along with the healthcare facilities in place on the cost of the insured population.

**Appendix II: organizational overview of the Dutch healthcare system**

**
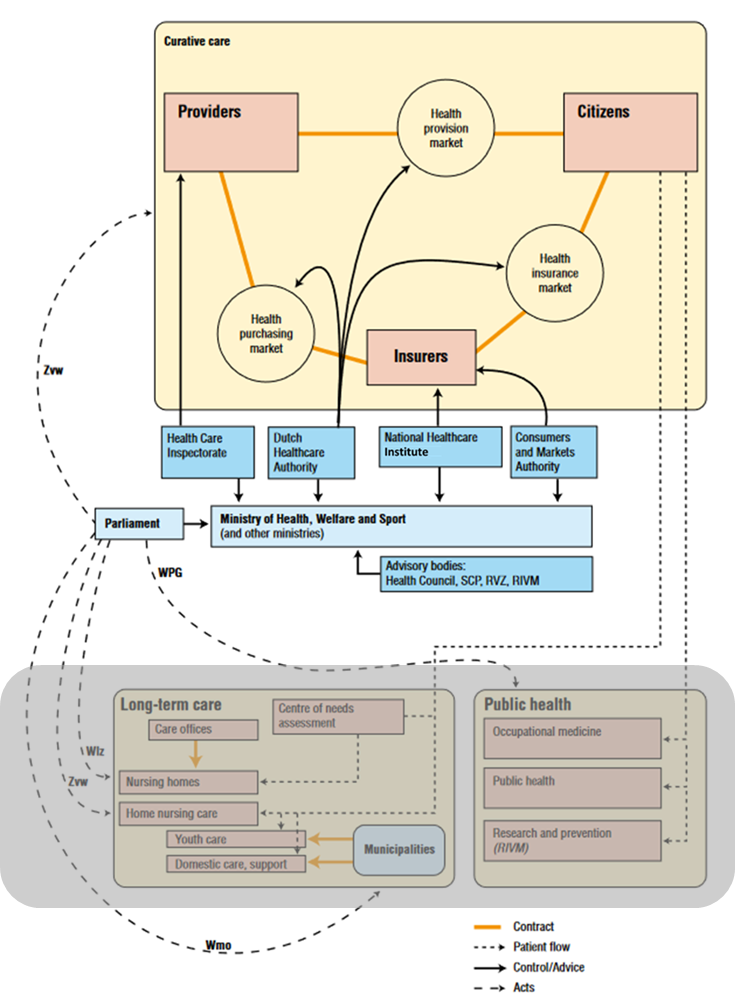
**

Figure A2-1. Adapted and adjusted from Kroneman et al. (2016). The grey box (long-term care and public health) is not relevant in the context of our study, as health insurers are only concerned with curative care. ZVW = zorgverzekeringswet (in English: Health Insurance Act). WLZ = wet langdurige zorg (in English: Longterm Care Act). Wmo = wet maatschappelijke ondersteuning (in English: ). WPG = wet publieke gezondheid (In English: Public Health Act). SCP = Social Cultural Planbureau. RVZ = Raad van Volksgezondheid en Zorg (in English: the Council of Public Health & Society). RIVM = Rijksinstituut voor Volksgezondheid en Milieu (in English: National Institute for Public Health and the Environment).

**Appendix III: topic list and interview guide**

The following topics were discussed during the interviews:

1. Insurer's stance and policy regarding shifting care from hospitals to general pracitioners;
2. Facilitating and hindering factors for this shift;
3. Developments in (and the relationships between) the national budgetary frameworks for general practitioner care and hospital care;
4. Heterogeneity of reimbursement structures between hospitals and general practitioners;
5. Incentivizes deriving from risk equalization;
6. Resources available to the insurer to effectuate the shift;
7. Possible solutions for discussed bottlenecks and the necessary parties for those solutions.

Interview guide

| **Interview phase** | **Topic** | **Description and questions** |
| --- | --- | --- |
| Introduction | - Context of the research - Objective of the research - Purpose of the interview | Emphasize that this is research for a dissertation on the shift in healthcare. It aims to provide insights from healthcare purchasers' perspective and map out their experiences. |
| Begin | - Name and position - Responsibilities at the insurer - How long have you been employed? |  |
| Case description |  | Policy-wise, there is a desire to shift care from the hospital to the general practitioner where possible. This shift can occur in multiple ways: by preventing hospital care at the GP and by referring patients back from the hospital to the GP more frequently. Research indicates that this shift does not always proceed smoothly. |
| Content | - Role of the insurer in the system/in integrated care - Biggest obstacles - What goes well, what doesn’t? - What solutions? By whom? | 1. To what extent is there policy on these various ways of shifting care between the GP and the hospital within your organization? 2. To what extent are concrete measures taken for this? What measures are they? 3. Is there a specific case you can use to illustrate this? 4. How does this policy affect your daily work? To what extent are you able to implement this policy in your daily work? 5. I assume you work within a certain budgetary framework, correct? If so, to what extent does this framework influence the extent to which this shift can be realized? 6. One of the factors influencing this framework is risk equalization. Recently, there have been several studies indicating that risk equalization does not always facilitate the shift to the GP. Are you aware of this, and do you recognize this from your daily work? 7. Previous research shows that the heterogeneous cost structure between GP care and hospital care can sometimes be a bottleneck for achieving the desired shift. For example, chronic conditions such as diabetes are paid for at the GP via bundled payments, but hospitals still have a production incentive. How do you experience this? 8. What is already going well in the shift of care from the hospital to the GP in your opinion? What evidence supports this? 9. What is not going well in the shift of care from the hospital to the GP in your opinion? What evidence supports this? 10. Where do you see the biggest obstacles? 11. For the shift of care from the hospital, the hospital must deliver less care. How do you achieve this? What tools do you have at your disposal? 12. For the shift of care from the hospital, the GP must deliver more care. How do you achieve this? What tools do you have at your disposal? 13. What factors, in your opinion, could contribute to a solution for the things that are not yet going well? 14. Which parties are needed for this? Who can achieve this, and who should take the lead in your opinion? |
| Conclusion | - Further progress of the project - Feedback on the interview - Feedback on results |  |

**Appendix IV: Coding tree**

Solutions

2.1. Means

2.1.1. Engage in conversation

2.1.2. Financially enforce

2.1.3. Best-effort obligation

2.2. Insurer's Approach

2.2.1. Market leader

2.2.2. Personal and regional

2.2.3. Position taken by the insurer

2.2.4. Mutual trust between insurer and healthcare provider

2.2.5. Short lines between buyers

2.3. Financial Incentive

2.3.1. Facilitating euros

2.3.2. Transformation funds

2.3.3. Innovative contracts for general practitioners

2.3.4. Corresponding claim

2.3.5. Reimbursement codes

2.3.6. Multi-year agreements

2.3.7. Insurer encourages general practitioners

2.3.8. Coherent agreement hospital - general practitioner

2.3.9. Sentiment on temporary funding

2.3.10. Care by general practitioners in MSZ funding

2.3.11. Subscription financing for general practitioners

2.4. National or Regional Policy

2.4.1. Regional agreements

2.4.2. Custom agreements

2.4.3. Zero growth hospital budget

2.4.4. National agreements

2.4.5. National policy as a driver

2.4.6. Policy from government

2.5. Personnel Organization

2.5.1. High healthcare demand in hospitals

2.5.2. Waiting list as a catalyst for substitution

2.5.3. Scalability

2.5.4. Scarcity of hospital staff

2.5.5. Practice support workers for general practitioners

2.5.6. Investing in primary care

2.5.7. Entrepreneurship in primary care

2.5.8. Specialization within general practice

2.5.9. Strength of general practice

2.5.10. ZBC ensures hospital efficiency

2.5.11. Commercial general practice

2.5.12. Digitization of general practice

2.6. Competition vs. Cooperation

2.6.1. Health insurers cooperate

2.6.2. Sufficient time as a prerequisite

2.6.3. Transmural agreements hospital - general practitioner

2.6.4. Consideration framework for insurer cooperation

2.7. Other

2.7.1. Telling the honest story

2.7.2. Learning from successes

2.7.3. Patient's wishes

Barriers

3.1. Other

3.1.1. Not cost-effective

3.1.2. Latent patients

3.2. Insurer's Approach

3.2.1. Small insurer

3.2.2. Insurer does not make an agreement about referrals back

3.2.3. Role of the insurer in the system

3.2.4. Substitution not as a goal

3.2.5. Fear of the unknown

3.2.6. Insurer as a bogeyman (advisor)

3.2.7. Aversion to change

3.2.8. Role of insurer in the system

3.2.9. Fear of missing something

3.3. Competency Profile

3.3.1. Core value of general practice

3.3.2. Competency profile

3.4. Competition vs. Cooperation

3.4.1. Competition among insurers

3.4.2. Not transferring patients

3.4.3. General practitioner cannot recall patient from hospital

3.4.4. They were always their patients (hospital)

3.4.5. Hospital does not support the movement

3.4.6. Half chain care without MSZ

3.4.7. Consultation culture

3.4.8. Lack of dialogue

3.5. Financial Incentive

3.5.1. Financial barriers

3.5.2. Financial incentives

3.5.3. Funding framework

3.5.4. Budgetary frameworks HAZ/MSZ

3.5.5. Risk equalization

3.5.6. Losing income

3.5.7. Deductible

3.5.8. Financial incentives

3.5.9. Transformation funds

3.5.10. Draw effect of consultation

3.5.11. Drop in the ocean regarding cost shift

3.5.12. Draw effect of intermediate care

3.5.13. Cashing agreements

3.5.14. Withdrawing money from hospital

3.5.15. Financial health of hospital

3.5.16. General practice funding from S3 to S1 can be a volume incentive

3.6. National or Regional Policy

3.6.1. Lead time

3.6.2. National policy

3.6.3. Barrier for insurer

3.6.4. Information asymmetry

3.6.5. For national policy, you need all insurers on board

3.6.6. Regional bottlenecks

3.6.7. Complicated NZa policy rules

3.7. Personnel Organization

3.7.1. Emergency care displaces planned hospital care

3.7.2. Capacity of general practitioners

3.7.3. Workforce planning for general practitioners

3.7.4. Shortage of general practitioners who own a practice

3.7.5. Scalability

3.7.6. Digital general practice

3.7.7. Barrier for hospital

3.7.8. Small volumes in primary care

3.7.9. Less strength of general practice

3.7.10. Layers within hospital

3.7.11. No urgency for change in general practice

3.7.12. Many hospitals in the region

3.7.13. Little volume growth in general practice

3.7.14. Scarcity of general practice staff

3.7.15. ZBCs take low complexity care from hospitals

3.7.16. Fragmentation of multidisciplinary care
